# Serotonin–dopamine polarity and social polarization

**DOI:** 10.3389/fpsyg.2026.1755161

**Published:** 2026-06-03

**Authors:** Joseph L. Giovannoli

**Affiliations:** Independent Researcher, Park Ridge, NJ, United States

**Keywords:** approach-avoidance, dopamine, Dunbar transition, evolutionary mismatch, group size, ideological divides, neurochemical predispositions, serotonin

## Abstract

This theoretical framework proposes that social polarization arises in part from an inherent neurochemical polarity in human groups. Variability in dopamine- and serotonin-linked approach-avoidance tendencies generates temperamental diversity that can be adaptive in small groups with dense, repeated interactions. When group size exceeds evolved cognitive limits on maintaining stable social relationships, these moderating mechanisms weaken, allowing unmoderated neurochemical differences to contribute to ideological division and reduced cross-group cooperation. The model integrates findings from neuroscience, behavioral genetics, evolutionary psychology, and cultural anthropology, emphasizing evolutionary mismatch between ancestral small-scale social environments and modern large-scale societies. Dunbar’s number (~150) and its layered structure (approximately 5, 15, 50, 150) serve as a heuristic mediator: dense inner-layer interactions moderate polarity through reciprocal trust and accountability, while larger, more anonymous networks amplify it. Cultural norms, social identity processes, and digital platforms further moderate these dynamics. The framework yields testable predictions, including reduced affective polarization in structured small-group settings, and suggests interventions such as facilitated small-group dialogs. It remains speculative and calls for preregistered, multi-method empirical validation.

## Introduction

Dopamine and serotonin are ancient monoaminergic systems that influence motivation, risk tolerance, and behavioral inhibition. Genetic variations (e.g., DRD4, 5-HTTLPR) and individual differences in these systems are associated with stable temperamental traits such as novelty seeking and harm avoidance (Ebstein et al., 1996; Munafò et al., 2008; Cloninger, 1987). In small groups, face-to-face interactions and reputation effects can balance these tendencies, supporting adaptive diversity. In larger, more anonymous settings, this balance weakens, potentially amplifying ideological divides.

This framework synthesizes neurochemical predispositions, cognitive constraints on group size, and evolutionary mismatch. It differs from motivated social cognition models or purely economic/institutional accounts by centering the interaction between neurochemical variability and relational scale. While associations exist between neuromodulator function and temperament or ideology, direct causal pathways remain probabilistic, task-dependent, and context-dependent (see Tables 1–3).

**Table 1:** Summary of selected cognitive/affective constructs, their canonical tasks, plausible neurochemical hooks, and representative peer-reviewed references.

Construct/Bias	Canonical task/measure	Mechanistic hook (interpretive)	Representative references
Optimism bias (gain-weighted belief updating)	Belief-updating from good vs. bad news	L-DOPA increases optimism bias by reducing learning from undesirable outcomes; it tilts expectations toward potential gains (dopamine).	Sharot T, Guitart-Masip M, Korn CW, Chowdhury R, Dolan RJ. (2012). How dopamine enhances an optimism bias in humans. Current Biology, 22(16), 1,477–1,481. https://doi.org/10.1016/j.cub.2012.05.053
Reward anticipation and risk preference	Monetary incentive delay (MID); risky choice tasks	Ventral striatum/ dopaminergic circuits engage during reward anticipation; correlate with risk-related behavior.	Knutson B, Cooper JC. (2005). Functional magnetic resonance imaging of reward prediction. Current Opinion in Neurology, 18(4), 411–417. https://doi.org/10.1097/01.wco.0000173463.24758.f6; Oldham S, Murawski C, Fornito A, Youssef GJ. (2018). A neuroimaging meta-analysis of the monetary incentive delay task. Human Brain Mapping, 39(9), 3,398–3,418. https://doi.org/10.1002/hbm.24184
Aversive learning/ behavioral inhibition	Fear conditioning; Go/No-Go; punishment learning	Serotonin modulates aversive processing and behavioral inhibition; it interacts with amygdala-centered fear circuits.	Dayan P, Huys QJM. (2009). Serotonin in affective control. Annual Review of Neuroscience, 32, 95–126. https://doi.org/10.1146/annurev.neuro.051508.135607; Bocchio M, McHugh SB, Bannerman DM, Sharp T, Capogna M. (2016). Serotonin, amygdala, and fear: Assembling the puzzle. Frontiers in Neural Circuits, 10, 24. https://doi.org/10.3389/fncir.2016.00024
Threat sensitivity (neural response)	Threat-of-shock; negative-valence images; risk task under fMRI	Amygdala engagement is robust in threat contexts; conservatives exhibited increased right amygdala activity in an fMRI risk task (task-dependent).	Schreiber D, Fonzo G, Simmons AN, Dawes CT, Flagan T, Fowler JH, Paulus MP. (2013). Red brain, blue brain: Evaluative processes differ in Democrats and Republicans. PLoS ONE, 8(2), e52970. https://doi.org/10.1371/journal.pone.0052970; Oxley et al. (2008) (Science) for physiological correlates of threat response.
Temperament heritability: novelty Seeking/Harm Avoidance	TPQ/TCI twin studies (adult Australian twin cohorts)	Genetic contributions to stable variation in NS/HA and RD (approximately54–61% of stable variance in a large twin sample).	Heath AC, Cloninger CR, Martin NG. (1994). Testing a model for the genetic structure of personality: A comparison of the personality systems of Cloninger and Eysenck. Journal of Personality and Social Psychology, 66(4), 762–775. https://doi.org/10.1037/0022-3514.66.4.762
Personality trait heritability (broad)	Meta-analysis across behavioral genetic studies	Personality traits exhibit moderate heritability across models and measures.	Vukasović T, Bratko D. (2015). Heritability of personality: A meta-analysis of behavior genetic studies. Psychological Bulletin, 141(4), 769–785. https://doi.org/10.1037/bul0000017
Political ideology heritability	Twin designs across democracies	Genetic influences contribute to political attitudes/ideology; effects are distinct from party ID.	Alford JR, Funk CL, Hibbing JR. (2005). Are political orientations genetically transmitted? American Political Science Review, 99(2), 153–167. https://doi.org/10.1017/S0003055405051579

It avoids ideologically specific causal claims and focuses on evidence-supported mechanisms.

**Table 2:** Factors contributing to polarization.

Factor	Evidence	Role
Psychological predispositions	Heritable links to brain structures (Oxley et al., 2008; Kanai et al., 2011)	Polarity engine from novelty-seeking to stability-prioritizing. Foundational for worldviews.
Elite cues and party sorting	Increasing ideological consistency since the 1970s (Mason, 2015)	Heighten issues, polarizing voters. Selective exposure via echo chambers (Prior, 2013; Bail et al., 2018). Institutional biases reduce opposing views.
Economic/social Shocks	Inequality heightens threats (Fiorina and Abrams, 2008; Hetherington and Weiler, 2009)	Triggers defensive responses, amplifying the engine.

**Table 3:** Comparison of polarization theories.

Theory	Core claim	Strengths	Limitations
Evolutionary mismatch	Large-group cognitive limits lead to self-sorting by risk tolerance; loss of huter-gatherer egalitarian leveling (Boehm, 1999)	Integrates biology; testable	Speculative; underemphasizes structures
Economic inequality	Inequality fuels tensions (Piketty and Saez, 2014)	Empirical; policy implications	Ignores cognitive drivers
Institutional polarization	Systems incentivize extremes (Fiorina and Abrams, 2008)	Explains politics; reform ideas	Overlooks predispositions
Cultural backlash	Changes provoke reactions (Norris and Inglehart, 2019)	Captures dynamics; cross-cultural	Less on biology/group size
This paper	Dopamine–serotonin neurochemical engine, mediated by Dunbar limits, drives mismatch polarization	Synthetic integration of neuro/psycho/cultural elements into a novel framework; predictions/interventions; unique mediation absent in Jost et al. (2003) or isolated studies	Speculative; needs validation

### Neurochemical foundations: dopamine and serotonin systems

Dopamine is centrally involved in reward anticipation, motivation, and exploration. Phasic dopamine bursts often signal positive prediction errors and invigorate approach behaviors, while tonic levels modulate baseline motivation and effort. Receptor subtypes matter: D1-like receptors (generally excitatory on direct-pathway medium spiny neurons) and D2-like receptors (inhibitory on indirect-pathway neurons) contribute to distinct aspects of decision-making and action selection. Dopamine effects on risk and learning are frequently dose-, task-, and context-dependent (Cools & D’Esposito, 2011; Norbury et al., 2013; St Onge and Floresco, 2009).

Serotonin modulates aversive processing, behavioral inhibition, and sensitivity to threat or uncertainty. It influences amygdala-centered fear circuits and can promote caution or punishment learning (Bocchio et al., 2016). Serotonergic and dopaminergic systems show functional interactions and, in some paradigms, opponent-like effects: dopamine biasing toward reward-seeking and optimism, serotonin toward inhibition and aversive learning. These interactions are not strictly push-pull; they depend on receptor profiles, brain regions (e.g., prefrontal cortex, striatum), and environmental context, and can be unified under affective, activational, and decision functions (Boureau and Dayan, 2011; Cardozo Pinto et al., 2025; Cools et al., 2011; Daw et al., 2002).

Individual differences arise from genetic polymorphisms, baseline neuromodulator tone, and experience. Twin studies indicate moderate heritability for novelty seeking/harm avoidance and broader personality traits (Heath et al., 1994; Vukasović and Bratko, 2015). These differences contribute to variation in risk tolerance and information processing biases (e.g., optimism bias linked to dopaminergic function; loss aversion and threat sensitivity associated with serotonergic pathways). Self-report measures such as the Behavioral Inhibition System/Behavioral Activation System (BIS/BAS) scales capture dispositional sensitivities to impending punishment (inhibition) versus reward (activation), which align with serotonergic and dopaminergic influences, respectively (Carver and White, 1994). Such variability is not deterministic and interacts with task demands and social context. Other neuromodulators (e.g., norepinephrine for arousal, oxytocin for bonding) may act as moderators or provide alternative pathways to approach-avoidance behaviors. Life-history trade-offs may further favor the evolution of such personality variation (Nettle, 2006; Wolf et al., 2007).

### Architecture of the proposed dopamine–serotonin polarity engine

The framework conceptualizes dopamine and serotonin as contributing to a polarity engine: configurations with relatively higher dopaminergic tone combined with lower serotonergic tone are associated with greater novelty seeking, reward anticipation, optimism bias, and risk-tolerant choices in many reward-related paradigms (Sharot et al., 2012). Conversely, configurations with relatively higher serotonergic tone and lower dopaminergic tone are linked to stronger harm avoidance, behavioral inhibition, sensitivity to negative outcomes, and more conservative decision-making under uncertainty or threat (Bocchio et al., 2016).

This engine operates at two levels:

- Within-person: dynamic balancing of approach and avoidance motivations in response to environmental cues.
- Between-person: stable temperamental differences that generate adaptive diversity within groups.

**Figure 1:**
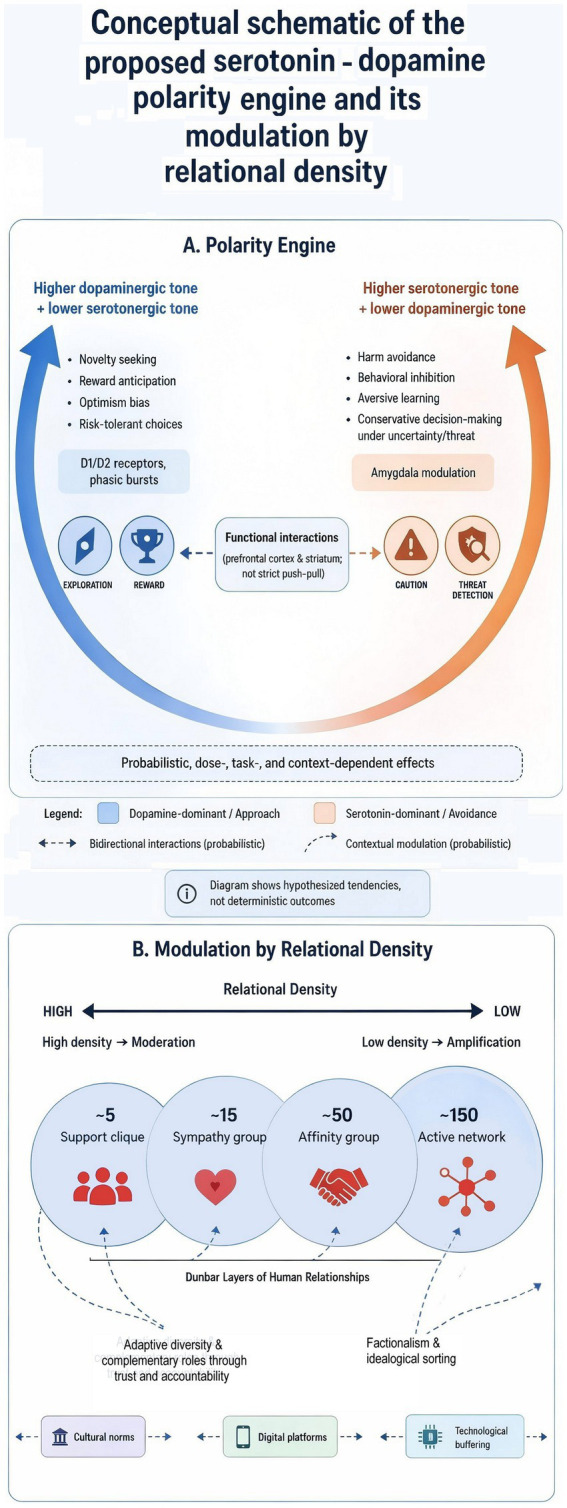
Conceptual schematic of the proposed serotonin-dopamine polarity engine and its modulation by relational density across Dunbar layers.

Inputs include genetic variants (e.g., DRD4, 5-HTTLPR), acute neuromodulation (pharmacological or environmental), and contextual factors. Outputs influence cognitive biases, risk preferences, and downstream social sorting. Effects are probabilistic and domain-specific; pharmacological or neuroimaging evidence shows task dependencies (e.g., dopaminergic enhancement of optimism bias in belief updating; serotonergic modulation of aversive Pavlovian processes). Receptor diversity (D1/D2), tonic versus phasic firing, and interactions via regions such as the prefrontal cortex and striatum add further granularity. The engine is not a simple linear driver but a flexible architecture whose influence on behavior is modulated by task, dose, and context (Cools et al., 2011; Robbins and Cools, 2014) (see Figure 1).

Panel A illustrates the polarity engine, with dopamine-related processes (higher tone associated with reward anticipation, exploration, optimism bias, and risk-tolerant choices in many paradigms) shown on one side and serotonin-related processes (higher tone associated with aversive learning, behavioral inhibition, harm avoidance, and conservative decision-making under uncertainty or threat) on the other. Dashed lines and notes indicate functional interactions in regions such as the prefrontal cortex and striatum, receptor subtype diversity (D1/D2), tonic versus phasic dynamics, and the probabilistic, dose-, task-, and context-dependent nature of effects. Panel B depicts the Dunbar layered model of relational density (~5 support clique, ~15 sympathy group, ~50 affinity group, ~150 active network) as a mediator: dense inner-layer interactions promote moderation and adaptive diversity through trust and accountability, while lower density in larger or more anonymous networks amplifies polarity toward factionalism and ideological sorting. Arrows and dashed pathways highlight hypothesized flows from neurochemical inputs to social outcomes. The diagram represents hypothesized tendencies within the theoretical framework rather than deterministic outcomes (Boureau and Dayan, 2011; Cardozo Pinto et al., 2025; Mac Carron et al., 2016).

### Linking neurochemical polarity and evolved cognitive biases

Dopamine-associated biases (e.g., optimism, overconfidence in gains) and serotonin-associated biases (e.g., loss aversion, negativity bias) align with evolved heuristics. In small groups with repeated interactions, these differences can foster complementary roles and adaptive problem-solving (Depue and Collins, 1999). This is consistent with humans’ evolved prosocial psychology, shaped by selection for friendliness and tolerance that supports cooperation despite individual variation in approach-avoidance tendencies (Hare, 2017). Beyond cognitive limits on stable relationships, anonymity and reduced accountability weaken natural moderation, allowing polarity to contribute to factionalism. Social identity processes and cultural narratives can further channel these tendencies into ideological divides. Neurocognitive and physiological correlates of political orientations (e.g., differences in conflict monitoring or threat sensitivity) provide complementary evidence that such temperamental variation may relate to ideological tendencies (Amodio et al., 2007; Kanai et al., 2011; Oxley et al., 2008). Economic stressors or institutional incentives may amplify rather than originate the underlying dynamics.

### Evolutionary mismatch and polarization dynamics

Human social cognition evolved in environments where groups rarely exceeded the cognitive capacity for dense, reciprocal relationships. This capacity reflects the social brain hypothesis, which links neocortex size to the cognitive demands of managing large social networks (Dunbar, 1998). Modern large-scale societies and digital platforms often exceed this, reducing opportunities for the repeated face-to-face interactions that historically moderated temperamental differences (Dunbar, 2016; Guess et al., 2023; Hare, 2017). Polarization—manifested as affective hostility and declining cooperation—can emerge when unmoderated neurochemical variability interacts with anonymity, echo chambers, and algorithmic amplification (Bail et al., 2018; Del Vicario et al., 2016). This is framed as an evolutionary mismatch rather than solely a failure of rationality or education.

### Variability in Dunbar’s number and its role as mediator

Dunbar’s number (~150 stable relationships) is a useful heuristic derived from primate neocortex scaling and human network data, with characteristic layered structure: approximately 5 (support clique), 15 (sympathy group), 50 (affinity group), and 150 (active network), sometimes extending to outer layers around 500 (Dunbar, 1992, 2021; Mac Carron et al., 2016; Powell et al., 2012; Stiller and Dunbar, 2007). The framework emphasizes the transition from dense inner layers—where repeated interactions and reputation maintain accountability—to more anonymous outer contacts, where moderation of polarity weakens.

Considerable variability exists across individuals, contexts, and cultures. Statistical critiques note wide confidence intervals in phylogenetic regressions, influences of ecology, diet, and institutions, and cultural flexibility in network maintenance (Dunbar, 2021). Collectivist norms may facilitate denser or more interdependent ties within layers, potentially buffering polarity through extended trust and conformity pressures, although direct cross-cultural network-size data are limited and subject to confounders (e.g., migration, measurement differences) (Chiao and Blizinsky, 2010; Kitayama et al., 2010; Markus and Kitayama, 1991). Individualistic contexts may allow greater expression of novelty-seeking but can exacerbate divides when meaningful connections are sparse. Technological tools expand superficial contacts without necessarily restoring dense reciprocal accountability (Dunbar, 2016).

The “Dunbar transition” is thus not a rigid universal threshold but a point of shifting relational density where the polarity engine’s balancing mechanisms become less effective.

### Cross-cultural studies and gene-culture coevolution

Gene-culture coevolution describes bidirectional influences between genetic variation and cultural practices (Boyd and Richerson, 2005; Cavalli-Sforza and Feldman, 1981; Mesoudi, 2011; Henrich, 2016; Richerson and Boyd, 2021). Studies have reported correlations between 5-HTTLPR short-allele frequency and collectivism, with higher short-allele prevalence in some collectivistic populations historically linked to heightened emotional sensitivity or harm avoidance (Caspi et al., 2003; Chiao and Blizinsky, 2010). Collectivist norms may buffer against negative outcomes associated with these variants by providing social support and conformity structures. However, replications and extensions show mixed results; environmental factors (pathogen prevalence, economic conditions, migration) often explain substantial variance, and associations with individualism–collectivism weaken or disappear after robust controls in some analyses. Dopamine-related variants (e.g., DRD4) have also been explored in relation to political or behavioral traits, with similarly modest and context-dependent effects (Ebstein et al., 1996; Munafò et al., 2008).

In this framework, cultural norms interact with neurochemical predispositions: collectivism may expand effective trust networks or promote conformity that dampens extreme expression of polarity, while individualism may amplify novelty-seeking but increase division in large-scale anonymous settings. These interactions remain correlational and require cautious interpretation to avoid overgeneralization or determinism. Preregistered cross-cultural designs with careful environmental controls are needed. Other approach/avoidance-relevant biochemicals (e.g., norepinephrine) may interact with or provide alternatives to the S/D engine.

### Derived hypotheses

*H1*: Individual differences in dopamine- and serotonin-linked traits (e.g., reward sensitivity, harm avoidance) are associated with variation in risk-related attitudes and ideological tendencies, though effects are probabilistic and mediated by context (Alford et al., 2005).

*H2*: Groups with denser, repeated interpersonal interactions (approximating inner Dunbar layers) exhibit lower affective polarization than larger, more anonymous settings; this is mediated by interpersonal trust and accountability.

*H3*: Affective polarization increases nonlinearly as relational density declines beyond levels supporting stable, reciprocal ties.

*H4*: Algorithmic mediation and one-off digital exposure amplify polarity compared with sustained face-to-face interaction (Bail et al., 2018; Guess et al., 2023).

These hypotheses are interdependent: relational scale moderates neurochemical influences, while cultural and technological factors act as additional moderators or amplifiers. Testing requires experimental group-size manipulations, network analyses, pharmacological challenges, and cross-cultural comparisons.

### Practical implications

Interventions that recreate conditions of dense, repeated interaction—such as structured small-group dialogs (15–30 participants) with facilitated cross-cutting discussion (Fishkin, 2009)—may restore moderation of polarity and reduce affective hostility. Network-based approaches that leverage “wide bridges” and peripheral seeding in moderately clustered groups can further facilitate the spread of cooperative norms across ideological divides (Centola, 2020). Educational approaches emphasizing awareness of non-volitional cognitive biases can promote intellectual humility. Platform design changes encouraging sustained rather than transient cross-group engagement warrant exploration. Lessons from collectivist contexts with strong normative buffering may inform institutional designs that expand cooperative opportunities without erasing beneficial diversity.

## Discussion

This framework reframes polarization as partly emergent from neurochemical diversity that is adaptive at small relational scales but becomes maladaptive when those scales are exceeded. It integrates micro-level neuromodulation, meso-level cognitive constraints on social networks, and macro-level cultural and technological moderators into a unified account.

### Positioning relative to existing theories

Unlike motivated cognition models focused on temperament-ideology links without explicit group-size mediation (Jost et al., 2003), or economic/institutional explanations that emphasize external shocks, this approach highlights why large-scale societies recurrently experience factionalism even absent acute stressors. It complements rather than displaces alternative accounts, positioning economic inequality, party sorting, or cultural backlash as potential amplifiers of an underlying polarity moderated by relational ecology (Hetherington and Weiler, 2009; Mason, 2015; Norris and Inglehart, 2019).

### Unresolved tensions and limitations

Direct causal links from specific neuromodulator function to ideological outcomes remain unestablished; evidence is largely correlational and context-dependent. Variability in network layers across individuals and cultures complicates universal claims. Cross-cultural patterns of polarization are heterogeneous, suggesting complex interactions with institutions and norms. Gene-culture associations, while intriguing, face replication challenges and require careful control for confounders. The framework is provisional and explicitly calls for empirical discrimination among competing models.

### Future directions

Preregistered studies should test hypotheses via:

- Randomized assignment to small- versus large-group deliberations.
- Neuroimaging and pharmacological modulation of dopaminergic/serotonergic function during ideological judgment tasks.
- Longitudinal network analyses tracking relational density and polarization.
- Multi-national designs examining cultural moderation while controlling environmental variables (Nosek et al., 2015).

## Conclusion

Social polarization has deep roots in the interplay between evolved neurochemical diversity and the cognitive limits of human social relationships. When moderated by dense, repeated interactions characteristic of small groups, this diversity supports adaptability. In larger, more anonymous contexts, it can contribute to division. By positioning layered relational constraints as a key mediator, the framework offers a biologically grounded yet culturally sensitive account and points toward scalable interventions that recreate conditions for effective moderation.

Progress depends on rigorous, interdisciplinary, preregistered research that refines or refutes these ideas. The ultimate goal is a more comprehensive science of both division and cooperation in complex societies.

## Data Availability

The original contributions presented in the study are included in the article/supplementary material, further inquiries can be directed to the corresponding author.
